# SG-APSIC1119: N95 mask concordance amongst female Muslim healthcare workers undergoing mask fitting with and without tudung

**DOI:** 10.1017/ash.2023.14

**Published:** 2023-03-16

**Authors:** Huiru Kui, Darius Beh, Xin Yi Lim, Brenda Ang, Bee Fong Poh

**Affiliations:** Tan Tock Seng Hospital, Singapore; Tan Tock Seng Hospital, Singapore; Tan Tock Seng Hospital, Singapore; Tan Tock Seng Hospital, Singapore; Tan Tock Seng Hospital, Singapore

## Abstract

**Objectives:** In August 2021, the Ministry of Health, Singapore revised the uniform policy in public hospitals to allow female Muslim staff, including nurses, to wear the tudung as an add-on to their uniforms. Institutions were advised that incorporation of the tudung should still align with current infection prevention guidelines. On May 2, 2021, in response to evolving evidence of SARS-CoV-2 transmission, our institution adopted the use of N95 masks for all HCWs in clinical settings. Prior to this revision in uniform policy, most female Muslim staff were mask fitted without tudungs. No existing international guidance recommends whether mask refitting of should be conducted with tudungs. As such, we looked at the N95 mask concordance for these staff undergoing mask fitting. **Methods:** Between November 1, 2021, and January 14, 2022, we mask fit-tested all new staff and refitted existing staff both with and without the tudung. We conducted qualitative fit-testing using their personal tudung, and we tested 2 models of N95 mask: 3MTM 1870+ and AIR+. When an HCW only passed the fitting of 1 or none of the models, additional N95 mask fit-testing was conducted with other available mask models according to our department’s existing workflow. **Results:** In total, 334 staff underwent N95 mask fitting. Overall, 326 (97.6%) passed with the same N95 mask models both with and without the tudung. The remaining 8 staff (2.4%) had passed 2 N95 mask models without the tudung but required a different N95 mask model while wearing the tudung. No staff required quantitative fit testing. **Conclusions:** N95 mask concordance for female Muslim staff undergoing fit-testing both with and without the tudung was high at 97.6%. Further evaluation of the 8 staff who did not show concordance could be retested using a quantitative fit-testing method.

